# Foreign Body in the Vagina of A Four-Year-Old-Girl: A Childish Prank or Sexual Abuse

**DOI:** 10.5812/ijhrba.10534

**Published:** 2014-06-21

**Authors:** Nahid Sakhavar, Batool Teimoori, Marzie Ghasemi

**Affiliations:** 1 Department of Obstetrics and Gynecology, Zahedan University of Medical Sciences, Zahedan, IR Iran

**Keywords:** Vaginal Discharge, Foreign Bodies, Sex Offenses

## Abstract

**Introduction:**

Foreign body in the vagina is a common cause of vaginal discharge, which may be either purulent or hemorrhagic.

**Case Presentation:**

This problem may produce symptoms or be asymptomatic for long periods of time and may result from ignorance, accident, malice, psychotic tendencies, attempts at sexual stimulation or sexual abuse. The current report presents the case of a girl that had inserted a foreign body in her vagina probably due to childish prank.

**Conclusions:**

The clinicians should always think of foreign bodies in the vagina in cases of chronic, antibiotic resistant vaginal discharge and lower abdominal pain especially in young girls.

## 1. Introduction

An extraordinary variety of foreign bodies may be found in the vagina, including safety pins, hair grips, pencils and small jam jars. The patient is often mentally retarded or a young child (1). Children insert toys, sweets, hairpins, etc. into the vagina mainly out of curiosity. Small pieces of toilet paper that find their way into the vagina are the most common cases (2, 3). Foreign bodies may be also inserted for various reasons, as articles of toilet and hygiene, by accident, as therapeutic agents to induce abortion or as contraceptive devices. Young children tend to explore all orifices and may place a variety of small objects in the vagina. So this is commonly a problem of children, who may insert a foreign body and not tell their parents. The child is finally often brought to the emergency department with a foul-smelling purulent discharge with or without vaginal bleeding. Vaginal foreign bodies in the adults may be a result of a psychiatric disorders or unusual sexual practices. Occasionally a tampon or pessary is forgotten or lost and causes discomfort and vaginal discharge (4).

## 2. Case Presentation

**Figure 1.**
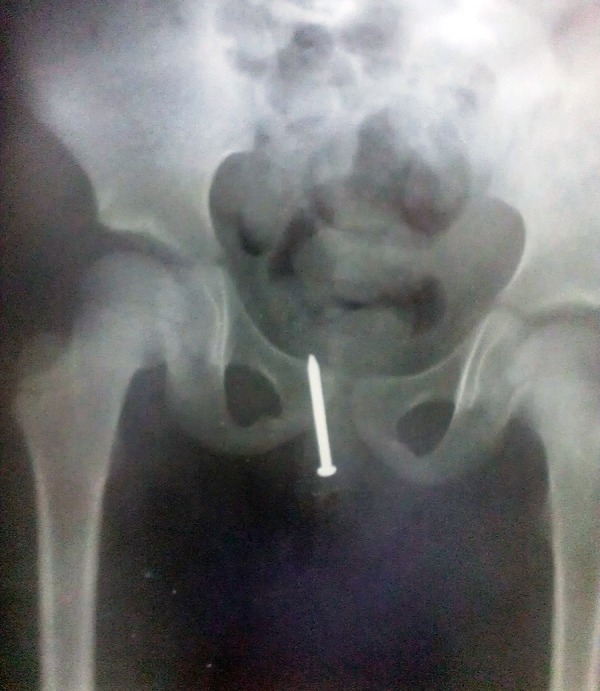
Pelvic X-Ray

**Figure 2.**
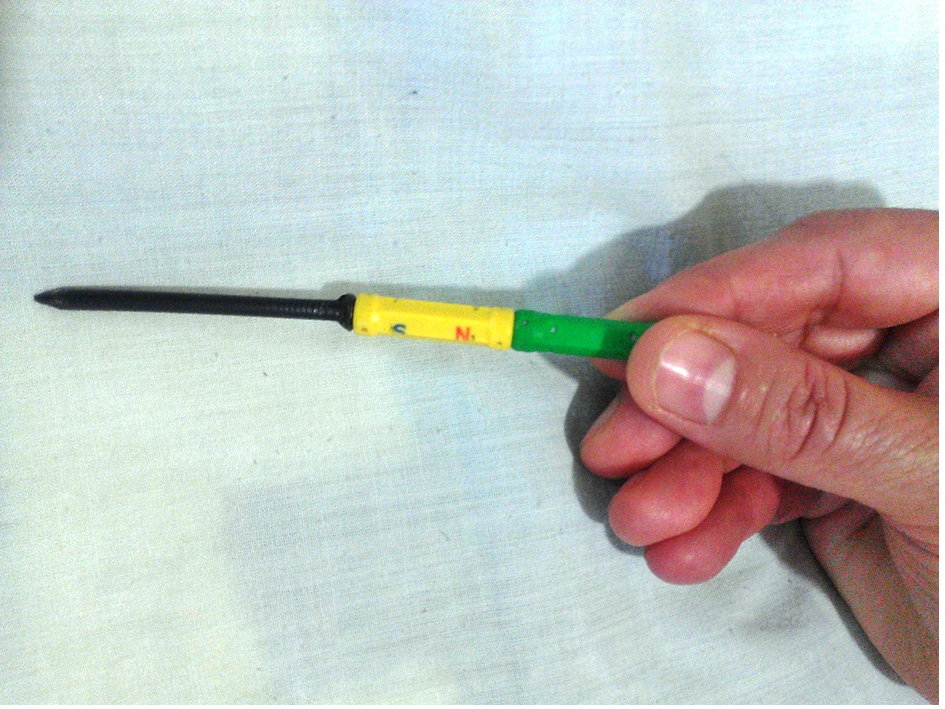
Magnetic Mag

A 4-year-old girl was brought by her mother from Saravan city to the emergency department of gynecology in Ali-Ibn-Abitaleb Hospital of Zahedan University of Medical Sciences in May 2012, with the complaints of a foreign body in her vagina and lower abdominal pain for eight hours. The clinicians did a pelvic X-Ray in Saravan city hospital that showed a big metal nail in the child`s vagina (Figure 1). In physical examination of the girl in frog-leg position and by tension of her vulvae labials major, a black point in depth of her vagina through the orifice of hymen was observed, it was the tail of a nail, the hymen was intact and her mother entreated that we discharge the nail without trauma to child hymen. Fortunately the cooperation of child was very good and we could discharge the nail by doing the TR (rectal exam with finger) and pushing the nail to midline of vaginal canal and extraction of nail out of vagina by use of a magnetic mag (Figure 2). In this case, it could not be established whether the nail had been inserted by the child herself or by another child or an adult.

## 3. Discussion

Vaginal foreign bodies are more commonly observed in children than in adolescent or adult women. Children may not be able to supply the history of an object placed in the vagina, however, some children will say that they have lost an object in their vagina. In addition to obtaining specific information about a possible vaginal foreign body, a health care provider will perform a general history and physical examination as well. It is appropriate for the health care providers to ask questions related to sexual activity and sexual or physical abuse. The presence of vaginal foreign body may be an indication of sexual abuse. Although this is not always the case, the possibility should be kept in mind while examining any child with vulvovaginal symptoms (4, 5).

Padmavathy et al. reported a three-and-half-year-old girl with a complaint of dysuria and foul smelling mucopurulent discharge from the vagina for three days. The child had lost her father 10 days ago. As there were many relatives at home on account of the recent bereavement, the grandmother was not sure of any sexual abuse. Local examination revealed a thick, foul smelling, and mucopurulent discharge at vulvar introitus. They presented two closed safety pins claimed to have been recovered from the child's vagina (6). Similar case has been reported by Simon et al. the long standing foreign body (fruit seed) in the vagina of a 13 -year-old girl that had caused chronic vaginal discharge, dense adhesion, and formation of granulation tissue in this young girl (7). McAllister also found a foreign body (flash light bulb) and vaginal stenosis in an 11-year-old girl who complained of intermittent foul-smelling vaginal discharge for nine years (8).

The effect of the object as a foreign body varies with its nature and shape; for example, perforation, abrasion, pressure necrosis and local vaginitis result in ulceration of the vaginal walls. This can involve neighboring structures to cause urinary and fecal fistulae. Ascending infection may lead to salpingitis and peritonitis. Rarely, neglected pessaries can cause severe ulceration of posterior fornix and later vaginal carcinoma (9, 10). The foreign body must be removed, which may be easy; although in young children a narrow illuminated endoscope may be needed. In the current case the good cooperation of the child lead to successful extraction of nail from her vagina by magnetic mag without laceration to her hymen. The clinicians should always think of foreign bodies in the vagina in cases of chronic, antibiotic resistant vaginal discharges and lower abdominal pain especially in young girls.
